# Digital Decision Aids to Support Decision-Making in Palliative and End-of-Life Dementia Care: Systematic Review and Meta-Analysis

**DOI:** 10.2196/71479

**Published:** 2025-06-23

**Authors:** Jie Zhong, Wei Liang, Tongyao Wang, Pui Hing Chau, Nathan Davies, Junqiang Zhao, Ho Nee Connie Chu, Chia Chin Lin

**Affiliations:** 1 School of Nursing Li Ka Shing Faculty of Medicine University of Hong Kong Hong Kong China (Hong Kong); 2 School of Nursing Nanjing Medical University Nanjing China; 3 Centre for Psychiatry and Mental Health Wolfson Institute of Population Health Queen Mary University of London London United Kingdom; 4 Dalla Lana School of Public Health University of Toronto Toronto, ON Canada; 5 School of Medicine and Dentistry University of Rochester Rochester United States; 6 Helping Hand Hong Kong China (Hong Kong)

**Keywords:** decision support tool, decision aid, palliative dementia care, Internet, meta-analysis

## Abstract

**Background:**

Making a care-related decision is a complex cognitive process. Patient decision aids could provide information on potential options about risks and benefits, incorporate individual values and preferences, and help people with dementia or their family carers make decisions about palliative and end-of-life care.

**Objective:**

This systematic review aimed to critically evaluate and synthesize evidence on the effectiveness of digital decision aids to support decision-making in palliative and end-of-life care for patients with dementia, their family carers, or clinicians.

**Methods:**

A systematic literature search in 4 health-related databases (PubMed, Embase, CINAHL, and Web of Science) was performed in September 2024. Articles were included if the study focused on the development and evaluation of a digital decision support tool on end-of-life dementia care, used an experimental design, and was available in full text in English. Studies using a nonexperimental design were excluded. The Cochrane Collaboration’s Risk of Bias Tool Version 2.0 or the Risk of Bias in Non-randomized Studies of Interventions Version 2.0 was used to assess risk of bias. Narrative synthesis and meta-analyses were performed to comprehensively summarize the technologies and outcomes of the decision aids.

**Results:**

The literature search across datasets identified a total of 1274 records. With an additional 5 records from citation searching and reference reviewing, a total of 20 articles were included, with 10 studies using data from randomized controlled trials (RCTs) and 10 pretest-posttest pilot studies. Technologies of visual aids, videos, web pages, and telehealth were reported in the included studies to support decision-making for end-of-life dementia care. Most decision aids focused on the decision about the primary goal of care (life-prolonging care, limited care, and comfort care), except for 1 visual aid focusing on the decisions about feeding tube placement and drug treatment for dementia. Most decision aids engaged both patients and their family carers. Pilot studies examining feasibility showed that most participants found these decision aids relevant to their needs and easy to use, and were able to complete the intervention sessions. Meta-analyses of 4 RCTs showed that video decision aids were effective in increasing the proportion of participants opting for comfort care (odds ratio 3.81, 95% CI 1.92-7.56) but inconclusive for the proportion of documented do-not-hospitalize orders (odds ratio 1.60, 95% CI 0.70-3.67), compared to the control group.

**Conclusions:**

Internet-based decision aids offer a feasible and acceptable approach to support the shared decision-making between patients, families, and clinicians. The included studies reported various outcome measures, including preferred goal of care, quality of palliative care, decision-making performance, and health care use. More large-scale RCTs are needed, and consistent outcome measures should be considered to evaluate the effects of end-of-life decision aids.

**Trial Registration:**

PROSPERO CRD42024621321; https://www.crd.york.ac.uk/PROSPERO/view/CRD42024621321

## Introduction

### Background

Dementia is a neurodegenerative syndrome in which the individual can experience loss of memory, problem-solving, and other cognitive abilities that interfere with daily living [1], as well as loss of independence, self-esteem, and autonomy [2]. The prevalence of dementia is increasing due to the rapid growth of an aging population worldwide. Approximately 50 million people are living with dementia, with an average new case of dementia every 3 seconds, which is projected to double every 20 years [3].

Life expectancy following a diagnosis of mild dementia ranges from 2 to 7 years, while for moderate to advanced dementia, it ranges from 1.5 to 2.5 years [4]. On the basis of the World Health Organization report in 2019, deaths due to dementia doubled between 2000 and 2016, which makes dementia the seventh leading cause of death worldwide [5]. Given that dementia is irreversible with no curative treatment, a palliative care approach can improve the quality of life among this population. However, many people with dementia receive palliative care less often and experience a high symptom burden at the end of life [6]. Previous studies indicate that people with advanced dementia have profound functional deficits that makes them dependent on full-time care, eventually leading to more intensive medical treatments against their preferences and wishes, such as cardiopulmonary resuscitation, placement on a ventilator [7,8], and nasogastric feeding tube placement, particularly in Asian countries [9]. Hence, there is an urgent need to engage in conversations with people living with dementia about their preferences regarding palliative and end-of-life care and document these decisions.

### Decision-Making in Dementia

Making a care-related decision is a complex cognitive process that requires substantial involvement of communication, attention, executive, and memory [10]. Healthy older people or older people at the early stage of dementia are cognitively capable of making decisions about care plans and medical treatments based on their own wishes and preferences. However, older people with advanced dementia may experience memory and language impairments that hinder their ability to remember new information and express their preferences [11]. As a result, depending on the national and local legislation, designated family carers may make end-of-life decisions on behalf of people with advanced dementia. However, substitute decision-making could trigger uncertainty and reactivity due to limited knowledge of the clinical condition and increased complexity in the care plan [12]. Given these challenges, advance care planning (ACP) emerges as a critical framework—an ongoing communication and decision-making process concerning goals and preferences of care between patients, family, and health care providers for future medical treatment and care before decisional capacity is lost [13,14]. By proactively engaging patients and their families in shared decision-making with clinicians, ACP aims to align future care with patient values. The development of structured decision support tools has become a key strategy to facilitate this process, particularly for navigating emotionally and medically complex choices [15].

### Digital Decision Aids

According to the International Patient Decision Aid Standards Collaboration, decision aids are defined as evidence-based tools designed to help people participate in decision-making about health care options [16]. Decision aids can support shared decision-making by providing evidence-based options with information on the probability of their benefits and risks [17]. Decision aids are most useful in situations where there is not 1 clear option and where the care pathway is highly dependent on individual preferences, values, and personal conditions [18]. Therefore, decision aids may be particularly helpful during end-of-life conversations. The traditional approach of using paper-based decision aids with verbal descriptions is constrained in realistically envisioning future disease states, maintaining consistency between different professionals, and accommodating varying levels of health literacy among patients [19]. Innovative technologies can facilitate the delivery of vivid (using visual images and videos), interactive (navigating the content and responding to interactive questions), dynamic (changing content according to user input and interaction), and tailored (evidence based on individual demographics or clinical conditions) decision support [20].

“Delivering patient decision aids on the internet” refers to the process of using the internet to provide some or all components of a patient decision aid to help individuals involved in the process of choosing between ≥2 appropriate care and life options [21]. The International Patient Decision Aid Standards Collaboration has included “delivering patient decision aids on the internet” as 1 of the 12 dimensions to assess the quality of patient decision aids [22]. According to a previous systematic review synthesizing decision aids to support decision-making in dementia care, 6 of the 10 included studies delivered paper-based decision aids, while the remaining 4 studies delivered video decision aids [23]. To our knowledge, there are currently no reviews that have explored the use of technologies (eg, video, visual aids, web-based decision aids, and mobile health apps) to support the development and delivery of decision aids on the internet for palliative and end-of-life dementia care.

### Objectives

In this review, we propose to synthesize digital decision aids to support decision-making in palliative and end-of-life dementia care. Specifically, this review aimed to summarize the following: (1) what are technologies, components, or procedures used in digital decision aids to support people with dementia or their family carers toward the end of life? and (2) what is the effectiveness of these decision aids on decision-making capacity, quality of palliative care, health care use, and related outcomes?

## Methods

### Overview

We conducted the systematic review and meta-analysis following the JBI Manual for Evidence Synthesis on conducting systematic reviews of effectiveness [24]. This review is reported using the principles of the PRISMA (Preferred Reporting Items for Systematic Reviews and Meta-Analyses) statement for reporting of systematic review and meta-analysis [25]. The PRISMA checklist was presented in Multimedia Appendix 1. The protocol was registered with PROSPERO (CRD42024621321).

### Search Strategy and Study Selection

A systematic literature review of 4 health-related databases (PubMed, Embase, CINAHL, and Web of Science) was performed in September 2024. There was no restriction on the search period. The search string was specified as Text Word [internet OR digital OR technology OR technologies OR visual OR web OR video OR audio OR media OR computer OR tablet OR electronic OR telecommunication OR telemedicine OR telephone OR television OR text messaging OR videoconferencing OR mobile phone] AND Text Word [informed choice OR decision-support* OR decision aids OR decision tree OR decision-mak* OR informed decision] AND Title/Abstract [end of life or eol or terminal care or palliative care or advance care planning or hospice care] AND Title/Abstract [memory disorder* or cognition or dementia or Alzheimer or dement* or cogni*]. The search strategy in each dataset is presented in Multimedia Appendix 2. The reference lists of included studies and relevant articles that cited included studies were hand-searched to identify additional eligible studies. After removing the duplicate results, titles and abstracts were screened based on the eligibility criteria independently by 2 reviewers (J Zhong and WL). All full texts were screened independently by 2 reviewers (J Zhong and WL). Any disagreement was resolved through discussion between the 2 reviewers, and a third reviewer (TW) was consulted when needed. Study screening and selection were conducted on Covidence (Veritas Health Innovation Ltd).

### Eligibility Criteria

Articles were included if they met the following criteria: (1) the focus was on the development and evaluation of a decision support tool on end-of-life dementia care; (2) the decision support tool used technologies (eg, videos, visual aids, and web pages); (3) the decision aid was aimed at older adults, family carers, or health care professionals; (4) the study used an experimental design (eg, randomized controlled trial [RCT], quasi-experimental, and pretest-posttest); and (5) the article was available in full text in English. Articles were excluded if they met any of the following criteria: (1) the decision support tool only provided information in texts without any visual aids or other technologies, (2) the decision was not related to end-of-life dementia care, (3) the full text was not available (eg, conference abstracts), (4) the article was a study protocol without reported results, or (5) the study used a nonexperimental design (eg, cohort study). The decision to focus on the experimental designs was guided by the primary aim to evaluate the effectiveness of digital decision aids, rather than examining their development or theoretical underpinnings.

### Data Extraction

Informed by the JBI Data Extraction Form for Systematic Reviews and Research Syntheses, 1 reviewer (J Zhong) extracted the data on the author, year, country, study design, sample characteristics, intervention (technology used in digital decision aids, components, or procedures), control, outcome measures, and effect of the intervention. For RCTs, data were obtained for both intervention and control groups. For pretest-posttest studies, data were extracted for the pretest-posttest sample. All data extraction was checked independently by the second reviewer (WL).

### Quality Evaluation

Quality of RCTs was evaluated using the Cochrane Collaboration’s Risk of Bias Tool (version 2.0) [26]. Each domain within the tool was assessed to have a low or high risk of bias. The quality of the pretest-posttest studies was evaluated with the Risk of Bias in Non-randomized Studies of Interventions Version 2.0 assessment tool. Each domain was judged to have a low, moderate, serious, or critical risk of bias. When all the domains were rated as having a low risk of bias, the study would receive an overall rating of low risk. If one domain of a study was considered as high risk of bias, the study was classified as having a high risk of bias overall. Two reviewers (J Zhong and WL) independently evaluated the quality of the included studies, and any disagreements were resolved through consultation with a third reviewer (TW) when needed.

### Data Synthesis and Analysis

Narrative synthesis was conducted based on the coding of the type of technologies (eg, visual aids, videos, and web pages) and outcomes (eg, preferred goal of care). Meta-analyses were conducted based on the extracted data in ReviewManager software (RevMan version 5.0) from the Cochrane Collaboration. Heterogeneity across studies was tested with *I*^2^ values, which ranged from 0% to 100%, with higher values indicating greater heterogeneity. When *I*^2^≥50%, which demonstrates substantial heterogeneity (*P*<.05), a random effects model was used; otherwise, a fixed effects model was used [27]. A meta-analysis was performed to determine the pooled effect of the intervention using standardized mean differences for continuous outcomes and odds ratio (OR) for dichotomous outcomes with 95% CIs when at least 2 same designed studies assessed the similar outcome.

## Results

### Description of the Included Studies

**Figure 1:**
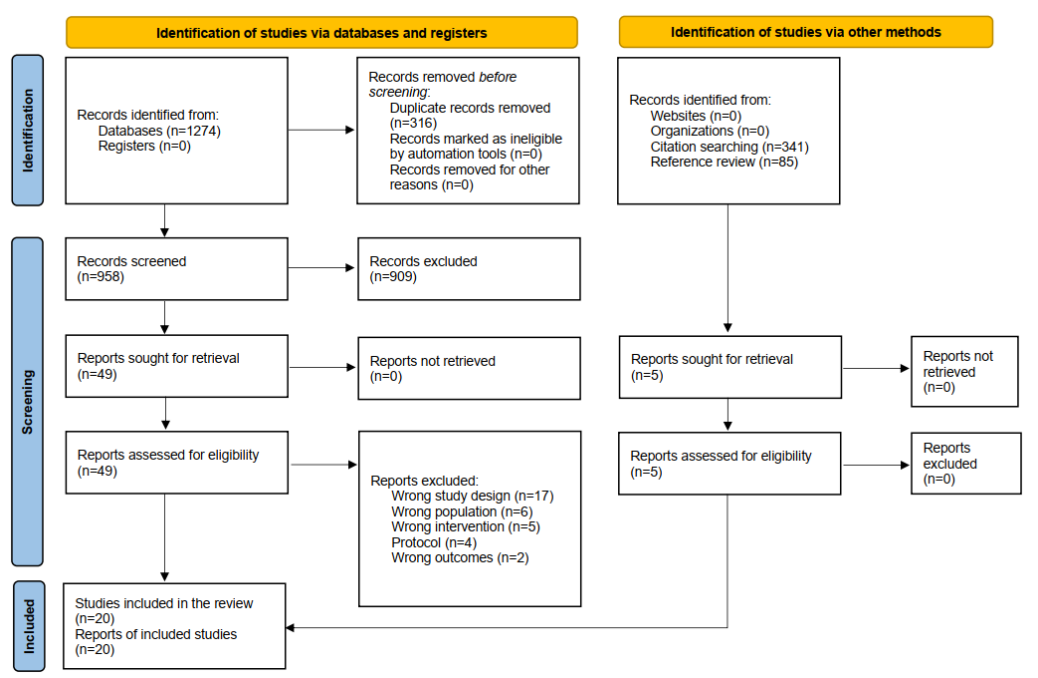
PRISMA (Preferred Reporting Items for Systematic Reviews and Meta-Analyses) flow diagram.

The searches yielded 1274 records. After removing 316 duplicates, 958 records were screened at the title and abstract stage, and 49 records were screened with full texts. With an additional 5 records from citation searching and reference reviewing, a total of 20 articles were included in the systematic review (refer to the PRISMA flow diagram in Figure 1). This review included 10 studies using data from RCTs [28-37] and 10 pretest-posttest or descriptive pilot studies [11,19,38-45]. Most studies were conducted in high-income countries, including the United States (17/20, 85%, including 3 secondary analyses of a cluster RCT, a pretest-posttest pilot study and an RCT testing the “My & My Wishes,” a pretest-posttest pilot study and an RCT testing a video decision aid, 4 studies testing a video decision aid across different settings, and 2 studies testing an interactive website), Australia (1/20, 5%), and the Netherlands (1/20, 5%), except for 1 in Taiwan (1/20, 5%).

Data extraction of the included studies is presented in Table 1. Most decision aids focused on the decision about the goal of care (life-prolonging care, limited care, and comfort care) in end-of-life dementia care, except for 1 visual aid focusing on 2 decisions—feeding tube placement and drug treatment for dementia [11]. Most decision aids targeted both patients and their family carers [28,34,36,38-45]. Some decision aids also included long-term care staff, case managers, or clinicians in the shared decision-making meetings [34,39,44,45].

**Table 1:** Characteristics of the included studies.

Study, study design, and country		Sample characteristics (sample size)	Intervention (components or procedures)	Control	Outcomes	Effect of intervention	
**Web-based decision aids**							
	Cardona et al [39], 2023; pilot usability testing; Australia	Older patients with dementia or chronic kidney diseases, informal caregivers (of patients with dementia), and clinicians (n=13 older patients or caregivers and n=12 clinicians)	Communicating Health Alternatives Tool: a web-based interactive app Components: Screening for short-term risk of death; Patient values and preferences; Treatment choices for chronic kidney disease and dementia.	None	Usability and acceptability: individual interviews		Clinicians, patients, and caregivers were generally accepting of its contents and format and supported its use in routine clinical practice.
	Behrens et al [38], 2022; pilot feasibility study; United States	People with advanced dementia living in nursing homes and their family surrogates (n=8 dyads); mean age: 75 y and mostly female (n=6)	“Our Memory Care Wishes”: a web-based decision aid tailored to dementia care Components: “My Goals of Care,” “My Supportive Decision Maker,” “My Dignified Day,” and “My Preferences for Future Medical Care”	None	Acceptability: Likert scales (eg, ease of use) and field observations through Zoom		Participants described ease of use, comfort with viewing, helpfulness for planning, and likelihood to recommend.
	Kotwal et al [43], 2021; pretest-posttest study; United States	Older adults with cognitive impairment and their family caregivers (n=20 dyads); mean age: 69.9 y for patients and 56.6 y for caregivers; 85% female patients and 70% female caregivers	PREPARE: a patient-centered, interactive website that uses video stories and modeling of ACP^a^ behaviors [46] Components: (1) choose a medical decision maker; (2) decide what matters most in life and for medical care; (3) decide on leeway for the surrogate decision maker; (4) communicate wishes with surrogates, clinicians, and other family and friends; and (5) ask the physician the right questions to make informed medical decisions.	None	Feasibility: 10-point ease-of-use scale, 5-point satisfaction scale, and 5-point feasibility scale ACP measures: 7-item general ACP knowledge and 15-item ACP engagement survey		Patients and caregivers rated PREPARE a mean of 8.6 and 9.4 on the 10-point ease-of-use scale, 4.7 and 4.7 on the 5-point satisfaction scale, and 4.9 and 4.8 on the 5-point feasibility scale, respectively. ACP engagement scores increased for 80% patients and 80% caregivers. Caregivers experienced increased knowledge from 3.8 to 4.7 and increased self-efficacy from 3.6 to 4.5 for ACP.
	Sudore et al [33], 2018; RCT^b^; United States	Primary care older patients with ≥2 chronic or serious illnesses (n=505 vs 481); mean age: 63.3 y	PREPARE plus an easy-to-read advance directive	An advance directive alone	New ACP documentation ACP engagement		ACP documentation rate difference: adjusted 43% versus 32% Increased ACP engagement rate: 98.1% versus 89.5%
	Span et al [44], 2015; pilot feasibility study; the Netherlands	People with dementia, informal caregivers, and case managers (n=4 patients, 12 informal caregivers, and 3 case managers); mean age: 77.5 y, 54.3 y, and 48 y, respectively	The DecideGuide: an interactive web tool that helps people with dementia, informal caregivers, and case managers make shared decisions Components: Chat enables users to communicate with each other, also from a distance; Deciding together assists decision-making step-by-step; Individual opinion enables users to give their individual opinions about dementia-related topics and individual circumstances.	None	Feasibility: interviews and field observations		Participants found the DecideGuide valuable in decision-making. The chat function seems powerful in helping members engage with one another constructively.
**Telehealth and EHR^c^ documentation**							
	Gabbard et al [41], 2023; pilot feasibility study; United States	Persons living with cognitive impairment and their care partners (n=163 patients and 45 care partners); mean age: 83.7 y; 68.2% female	ACPWise: an interface that allows the primary care provider team (physician, physician assistant, nurse practitioner, or social worker) to document conversations in a standardized manner using discrete data elements within the EHR and allows for free-text comments and responses. Procedures: the provider scheduled the telehealth visit and covered topics of assessing disease understanding and prognosis; reviewing current goals, values, and concerns; reviewing any unacceptable states, future care preferences, and preferred surrogate decision maker; and asking whether they have any ACP documents.	None	Feasibility: completion rate, conversation documentation, and use of ACP billing codes		76% completed the telehealth intervention. Among participants who completed the intervention, conversation documentation and use of ACP billing codes was 100% and 96%. Adoption at clinic level was 50%, while 75% of providers within these clinics participated.
**Visual aids**							
	Chang and Bourgeois [11], 2020; pretest-posttest; United States	Residents with a diagnosis of dementia in facilities (n=100); mean age: 87.10 y; 90% female	The intervention includes (1) 2 medical vignettes; (2) 2 sets of decisional aids with pictures and sentences corresponding to each vignette; and (3) a questionnaire that encompasses a record for, a scoring form, and scoring criteria. Decisional scenarios included (1) feeding tube placement for dysphagia: whether to insert a feeding tube when one has difficulty swallowing and (2) drug treatment for dementia: whether to take dementia medications with unpleasant side effects.	Verbal condition	Decisional performance evaluated by external judges by listening to each sample through headphones on 4 legal standards (Understanding, Expressing a Choice, Reasoning, and Appreciation)		Pretest-posttest score difference: 84.3 (SD 7.39) versus 49.1 (SD 8.87); Cohen d=4.44
	Huang et al [42], 2020; pretest-posttest; Taiwan	Persons diagnosed with mild cognitive impairment or mild dementia and their caregivers (n=40 patients and 38 family caregivers); mean age: 77.5 y in patients and 56.9 y in caregivers; 53% female patients and 85% female caregivers	An ACP manual, titled, Be My Own Master: talk about ACP for dementia (descriptions about the symptoms of end-stage dementia and the common EOL^d^ life-sustaining medical treatments, such as CPR^e^, machine ventilation, tube feeding, intravenous infusion, and antibiotics). The manual also provided details about the benefits and risks of the treatments, as well as the process of establishing ACP and the regulations involved.	None	Knowledge of end-stage dementia and ACP Decisional conflict		Difference in knowledge of end-stage dementia treatment: 6.38 (SD 4.16) versus 8.75 (SD 4.74); Cohen d=0.5 Difference in knowledge of ACP: 2.95 (SD 3.49) versus 5.33 (SD 4.20); Cohen d=0.6 Difference in decisional conflict scores: 49.88 (SD 21.03) versus 35.11 (SD 16.62); Cohen d=−0.8
**Video decision aids**							
	McCreedy et al [30], 2022; secondary analysis of a cluster RCT; United States	3902 long-stay residents with advanced illness (1485 treatment and 2417 control) and 2215 short-stay residents with advanced illness (873 treatment and 1342 control)	PROVEN^f^: 5 previously created videos (6- to 10-min long) offered in English or Spanish (General Goals of Care, Goals of Care for Advanced Dementia, Hospice, Hospitalization, and ACP for Healthy Patients) Procedures: program champions (typically social workers) showed the videos to the resident or their proxy at 7 days of admission or readmission, every 6 months for long-stay residents, when decision-making arose on a topic for which there was a specific video, on a significant change in clinical status, and special circumstances when GOC^h^ are considered.	Usual ACP procedures	Proportion of residents acquiring a new DNH^g^ order in the EHR Proportion of residents having at least 1 hospitalization		During the follow-up (12-mo for long-stay residents and 100 d for short-stay residents) Proportion difference in DNH order: 9.3 (95% CI 5.0-13.6) versus 4.2 (95% CI 2.1-6.2), average marginal effect of 5.0 (95% CI 0.3-9.8) in long-stay residents; 8.0 (95% CI 4.6-11.3) versus 3.5 (95% CI 1.5-5.5), average marginal effect of 4.4 (95% CI 0.5-8.3) in short-stay residents Proportion difference in hospitalization: 28.4 (95% CI 25.3-31.5) versus 28.8 (95% CI 25.8-31.7), average marginal effect of −0.4 (95% CI −4.7 to 3.9) in long-stay residents; 39.9 (95% CI 36.1-43.6) versus 38.3 (95% CI 35.6-41.0), average marginal effect of 1.5 (95% CI −3.1 to 6.2) in short-stay residents
	Moyo et al [32], 2022; secondary analysis of a cluster RCT; United States	2848 decedents among long-stay residents with advanced illness (923 intervention and 1925 control)	PROVEN	Usual ACP procedures	Proportions of 90-d hospital transfers, multiple hospital transfers, and late transitions		Proportion differences (intervention—control): 90-d hospital transfers (−1.71, −3.21 to −0.09), multiple hospital transfers (−0.83, −1.71 to 0.14), and late transitions (−2.22%, 95% CI −5.29% to 1.26%)
	Loomer et al [29], 2021; secondary analysis of a cluster RCT; United States	2538 and 5290 short-stay patients with advanced illness (intervention and control); 23,303 and 50,815 short-stay patients without advanced illness (intervention and control)	PROVEN	Usual ACP procedures	Hospital transfer in 1000 person-days alive Proportion of patients experiencing ≥1 hospital transfer, ≥1 burdensome treatment, and hospice care enrollment		Marginal rates of hospital transfer per 1000 person-days alive: short-stay patients with advanced illness (12.3, Marginal rates of hospital transfer per 1000 perso11.6-13.1) versus 13.2, 12.5 to 13.7), marginal rate difference: −0.8 (−1.7 to 0.2); short-stay patients without advanced illness (9.5, 9.3 to 9.8 versus 9.3, 9.1 to 9.5), marginal rate difference: 0.2 (−0.1 to 0.6) The proportions of patients who experienced ≥1 hospital transfer, ≥1 burdensome intervention, or hospice care enrollment did not differ significantly between 2 groups in both short-stay patients with and without advanced illness.
	Mitchell et al [31], 2020; cluster RCT; United States	360 nursing homes (119 intervention vs 241 control); 4171 and 8308 (intervention and control) long-stay residents with advanced illness; 5764 and 11,773 (intervention and control) long-stay residents without advanced illness	PROVEN	Usual ACP procedures	Hospital transfer Proportion of patients experiencing ≥1 hospital transfer, ≥1 burdensome treatment, and hospice care enrollment		During the 12-mo follow-up Hospital transfer rate difference: 3.7 (95% CI 3.4-4.0) versus 3.9 (95% CI 3.6-4.1); −0.2 (95% CI −0.5 to 0.2) in residents with advanced illness; 0.0 (95% CI −0.3 to 0.2) in residents without advanced illness Proportion differences: ≥1 hospital transfer, ≥1 burdensome intervention, or hospice care enrollment did not differ significantly between the 2 groups.
	Towsley et al [45], 2020; descriptive pilot study; United States	Nursing home residents without severe cognitive impairment, families, and staff (n=20); mean age: 72.2 y	Personalized “Me & My Wishes” video was recorded and structured into 4 parts: About Me, Preferences for Today, Preferences for Medical Intervention and End-of-Life Care, and Afterthoughts Procedures: study coordinator facilitates video-recording conversations that cover learning about the resident as a person as well as their preferences for everyday care and activities, medical intervention, and psychosocial preferences near EOL and after death preferences. Residents approve their personalized video, which is then shared with the care plan team and resident-identified family members or friends.	None	Feasibility: enrollment, completing and sharing the video, and scales		20 residents recorded the video, 18 residents shared their videos with family or staff, and 14 residents made positive comments about the video conversation. Residents described the video as honest, effective, and eye-opening about themselves, and they stated that it reflected their point of view. On a scale of 1 to 7 (lower is better), mean ratings were 2.0 (family) and 1.3 (staff) for communication quality for how the video conveyed the resident’s preferences for daily care; 1.9 (family) and 1.2 (staff) for communicating preferences for EOL. Both family and staff reported increased knowledge about resident preferences for daily and EOL care.
	Towsley et al [34], 2022; randomized waitlist control; United States	Long-term care residents living with dementia, families, and staff (n=36 residents, 50 families, and 38 staff); mean age of 78.4 y and 61.1% female residents; mean age of 60.7 y and 72% female family members; mean age of 43.2 y and 84.2% female staff	Personalized “Me & My Wishes” video	Usual care	Family-resident concordance of EOL treatment preferences Family-resident concordance of near-EOL psychological preferences Staff-resident concordance of EOL treatment preferences Staff-resident concordance of near-EOL psychological preferences		Time of sharing the video and 90-d follow-up: For EOL treatment preferences, resident-family concordance increased at intervention (time of sharing the video, β=.21, 0.11 to 0.30), but dropped back to baseline rates at follow-up (β=.07, 95% CI −0.08 to 0.22). For EOL psychosocial preferences, resident-family concordance did not show significant increase at intervention (β=.09, 95% CI −0.01 to 0.19) and at follow-up (β=−.03, 95% CI −0.19 to 0.13). Resident-staff concordance in EOL treatment preferences increased at intervention (β=.35, 95% CI 0.21-0.48) and at follow-up (β=.40, 95% CI 0.21-0.58). Resident-staff concordance in EOL psychosocial preferences increased at intervention (β=.29, 95% CI 0.12-0.46) but dropped back to baseline at follow-up (β=.08, 95% CI −0.15 to 0.30).
	Hanson et al [28], 2017; cluster RCT; United States	Nursing home residents and their designated family decision makers (n=151 vs 151); mean age of residents was 86.5 y and 81.5% were women; mean age of family decision makers was 63 y and most were daughters or daughters-in-law.	An 18-min video decision aid about GOC choices in advanced dementia. Procedures: a research staff ensured the decision maker watched the video during their initial study visit and scheduled a structured nursing home care plan meeting to address GOC.	Usual care	Family-rated QOC^i^ with nursing home staff Concordance with clinicians on GOC ACP problem score Quality of palliative care at 6 mo and at 9 mo or death		At 3 mo Score difference on QOC questionnaire: 6.0 (SD 1.8) versus 5.6 (SD 2.0) At 9 mo Proportion difference on concordance with clinicians: 88.4% versus 71.2%. Score difference on symptom management and satisfaction with care did not differ between groups. Medical Orders for Scope of Treatment order set: 35% versus 16%
	Einterz et al [40], 2014; pretest-posttest; United States	Nursing home residents and their surrogate decision makers (n=18 dyads); mean age of 67 y in residents and 90 y in surrogates; 56% female residents and 83% female surrogates	Same as above	None	Feasibility (89% of family decision makers thought the decision aid was relevant to their needs) Knowledge of advanced dementia QOC Number of palliative care domains addressed in the care plan		Knowledge score difference: 12.5 versus 14.2 at 3 mo QOC score difference: 6.1 versus 6.8 Proportion difference on concordance with clinicians: 50% versus 78% Difference in domain numbers: 1.8 versus 4.3
	Volandes et al [35], 2023; RCT; United States	Patients aged ≥65 y from 14 hospital units (n=6023 vs 4779)	Palliative care educators facilitating GOC conversations using a library of brief, certified video decision aids that addressed a range of topics (GOC, CPR, hospice, palliative care, time-limited trials, Alzheimer disease and related dementias, and COVID-19)	Usual palliative care	Proportion of GOC documentation Proportion of documented conversations of goals, limitations of life-sustaining treatment, and palliative care		Immediately after the intervention Difference in proportion: 62.2% (3744/6023) versus 50.1% (2396/4779) Difference in proportion of documented conversations: 59.1% (3562/6023) versus 47.2% (2258/4779) in goals, 32.9% (1979/6023) versus 26% (1242/4779) in limitations of life-sustaining treatment, and 34.3% (2067/6023) versus 14.6% (700/4779) in palliative care
	Volandes et al [36], 2009; RCT; United States	Patients aged ≥65 y from 4 primary care clinics; mean age: 75 y; 58% women; 9% had diagnosis of dementia (n=94 vs 106)	Listening to the verbal narrative, followed by watching a 2-min video depicting a patient with advanced dementia	Listening to a verbal narrative describing advanced dementia	Preferred GOC: life-prolonging care, limited care, or comfort care Knowledge of advanced dementia evaluated by 5 true or false questions (0 to 5)		Immediately after the intervention Difference in percentage of patients choosing comfort care: 22% (95% CI 11%-34%), unadjusted odds ratio: 3.5 (95% CI 1.7-7.1) Mean difference in knowledge score: 4.5 (SD 1.0) versus 3.8 (SD 1.3)
	Volandes et al [37], 2009; RCT; United States	Patients aged ≥65 y from 2 geriatric clinics and their surrogates (n=8 vs 6)	Same as above	Same as above	Preferred GOC (opting for comfort care) Patient-family concordance in GOC (comfort care) Knowledge of advanced dementia		Immediately after the intervention (intervention vs control) Opting for comfort care: 100% versus 50% Patient-family concordance: 100% versus 16.7% The change in knowledge scores: 2.5 (SD 1.4) versus 2.0 (SD 1.3)
	Volandes [19], 2007; pretest-posttest; United States	Older patients in primary care clinics (n=120)	Same as above	None	Preferred GOC		Before and after proportion difference: 60 (50%) versus 107 (89.2%) opting for comfort care

^a^ACP: advance care planning.

^b^RCT: randomized controlled trial.

^c^EHR: electronic health record.

^d^EOL: end-of-life.

^e^CPR: cardiopulmonary resuscitation.

^f^PROVEN: Pragmatic Trial of Video Education in Nursing Homes.

^g^DNH: do-not-hospitalize.

^h^GOC: goal of care.

^i^QOC: quality of communication.

### Quality Appraisal

The results of the quality appraisal are presented in Multimedia Appendix 3 [11,19,28-45]. Allocation concealment was rated as unclear in 1 RCT [32]. Three RCTs were rated as having a high risk of bias for blinding of participants and personnel, as the interviewer was involved in delivering part of the intervention (eg, providing a verbal narrative) [34,36,37]. Blinding of outcome assessment was rated as high risk in 2 RCTs, as they reported that the data assessor was not blinded to the group allocation [36,37]. Overall, 10 pretest-posttest pilot studies were evaluated using the Risk of Bias in Non-randomized Studies of Interventions Version 2.0. Three studies were rated as moderate risk during the selection of participants into the study because they either recruited participants from a single center or did not report the procedure of participant recruitment with a small sample size [38,39,44]. Three studies were rated as unclear bias regarding deviations from intended interventions because they did not report on the participants’ adherence to the intervention [38,39,44].

### Technologies Used in Digital Decision Aids

Visual aids, videos, web pages, and telehealth were applied in the identified digital decision aids to support decision-making for palliative and end-of-life dementia care.

#### Visual Aids

External visual supports in the form of graphs and pictures were reported to compensate for the decreased level of communication and cognitive abilities in people with dementia. Picture-text visual aids are developed to support ACP conversations and decision-making about end-of-life dementia care [11,42]. The visual aid described the symptoms of end-stage dementia, common life-sustaining treatments used at the end of life, and details about the benefits and risks of the treatments [42]. With verbal guidance, participants are presented with pictures and written text about the potential treatment for easy interpretation [11].

#### Video Decision Aids

The most used technology was video decision aids. In the United States, video decision aids for dementia were developed by Volandes et al [19,36,37], Hanson et al [28], Einterz et al [40], and Mitchell et al [31]. The video library addressed a range of topics, including principal features of advanced dementia (incurable progression, inability to communicate, inability to ambulate, and inability to feed themselves), goals of prolonging life, supporting function, or improving comfort; treatments consistent with each goal (eg, cardiopulmonary resuscitation and hospitalization); hospice; and palliative care [19,28,31]. Interventionists (such as social workers, nurses, and research staff) facilitated participants to watch the video during the initial study visit, within 7 days of admission or readmission, every 6 months for long-stay residents, when a decision-making need arose on a topic for which there was a specific video, on a significant change in clinical status, and under special circumstances when goals of care (GOC) were considered (eg, family visiting) [31]. After watching the video, a structured discussion with clinicians (such as the nursing home care team) was scheduled to incorporate decisions into daily care [28]. Feasibility results across studies supported that 88% of patients found the video “very helpful” or “somewhat helpful” [37] and 89% of family decision makers thought the video was relevant to their needs [40].

Except for the prerecorded videos shared with patients and their families, another approach was to record a personalized video regarding the preferred goal of care. Towsley et al [34,45] structured a personalized “My & My Wishes” video for long-term care residents with dementia and shared it with their family and staff. Facilitated by a study coordinator based on a conversation guide, the video was structured into 4 sessions: about me, preferences for today, preferences for medical intervention and end-of-life care, and afterthoughts. During the care plan meeting, the resident, family, and care team would view the video together and clarify or reiterate preferences most important to the resident. Residents described the video as honest, effective, and eye-opening about themselves, and they stated that it reflected their point of view [45].

#### Web-Based Decision Aids

Web-based decision aids offer several benefits, including the flexibility about the individual’s preferred time and place, their relatively anonymous use, and their ability to record personalized activities and information. Four websites were identified to help people with dementia, family carers, and clinicians make informed, shared decisions. First is the DecideGuide. It has three primary functions: (1) chat (enables users to communicate with each other from a distance), (2) deciding together (assists decision-making step-by-step), and (3) individual opinion (enables users to give their individual opinions about dementia-related topics and individual circumstances) [44]. Patients found the DecideGuide valuable in decision-making, particularly they found the chat function to be powerful in helping members in their dementia care networks engage with one another constructively. Second is the PREPARE website. It includes five steps for shared decision-making: (1) choose a medical decision maker; (2) decide what matters most in life and for medical care; (3) decide on leeway for the carer decision maker; (4) communicate wishes with carers, clinicians, and other family and friends; and (5) ask the physician the right questions to make informed medical decisions [33,43]. Patients and carers rated PREPARE with high scores in ease of use, satisfaction, and feasibility. The third one is “Our Memory Care Wishes.” It has 4 tailored modules for dementia: “My Goals of Care,” “My Supportive Decision Maker,” “My Dignified Day,” and “My Preferences for Future Medical Care.” Participants reported that the modules were easy to use, comfortable to view, helpful for planning, and that they would likely recommend them [38]. The last one is Communicating Health Alternatives Tool, which was designed to be compatible with hospital medical records software to facilitate patient-centered decision-making across health settings. Components included screening for short-term risk of death, patient values and preferences, and treatment for chronic kidney disease and dementia. Clinicians, patients, and carers were generally accepting of its contents and format and supported its use in routine clinical practice [39].

#### Telehealth and Electronic Health Record Documentation

Telehealth could be applied to help patients voice their preferences and document their decisions in the electronic health record (EHR) system. The TeleVoice was a telehealth intervention for serious illness conversations via video or telephone. Health care providers assisted persons living with cognitive impairment in discussing their current goals, values, and future medical preferences, while facilitating documentation within the EHR. Components of the telehealth visit included assessing disease understanding and prognosis; reviewing current goals, values, and concerns; reviewing any unacceptable states, future care preferences, and preferred carer decision maker; and asking whether they have any ACP documents. An interface, ACPWise, allowed providers to document conversations in a standardized manner and allowed for free-text comments and responses. Of the 163 eligible persons approached, 76% completed the telehealth intervention, and 45 care partners agreed to participate. Adoption at the clinic level was 50%, while 75% of the providers within these clinics participated. Among participants who completed the intervention, conversation documentation and use of ACP billing codes were 100% and 96%, respectively [41].

### Effectiveness of Decision Aids

#### Preferred Goal of Care

Three studies conducted by Volandes et al [19,36,37] used the preferred goal of care (life-prolonging care, limited care, and comfort care) as the primary outcome of the video decision aid. The research demonstrated that after watching the video decision aid, there was an increase in the proportion of participants choosing comfort care. The studies included a pretest-posttest pilot study with 120 patients from primary care clinics, an RCT with 200 older patients from primary care clinics, and another RCT with 14 pairs of older patients and their caregivers from geriatric clinics. The results indicated that the video decision aid facilitated older patients in making a more stable decision in favor of comfort care, with fewer participants changing their preferences after 6 weeks. These consistent findings suggest that video decision aids can assist older patients in making informed and stable decisions regarding their care preferences.

#### Goal of Care Concordance

The studies by Towsley et al [34], Hanson et al [28], and Einterz et al [40] focused on concordance with the goal of care between patients, family members, and staff or clinicians as their primary outcome. Concordance occurs when both endorsed the same goal as the “best goal to guide care and medical treatment” and “top priority for care and medical treatment.” In a study by Hanson et al [28], involving nursing home residents with advanced dementia and their family decision makers, comfort care became increasingly the primary goal of care over time for both groups. Compared to the control group, goal concordance did not differ at 3 months but showed a difference by 9 months or death. In the pretest-posttest study, family decision makers showed an increased concordance with clinicians on the primary goal of care. In a study by Towsley et al [34], the “My & My Wishes” program extracted care preferences from personalized videos for each patient, leading to increased concordance between residents, family members, and staff on end-of-life treatment and psychosocial preferences. While the video decision aids improved concordance in the short term, the long-term effect on maintaining concordance was found to be unstable in these studies.

#### Quality of Palliative Care

The studies by Hanson et al [28] and Einterz et al [40] assessed the quality of palliative care by measuring family-rated quality of communication, Symptom Management at the End of Life in Dementia, Satisfaction with Care at the End of Life in Dementia, ACP problem, and palliative care treatment plan domain. In a cluster RCT involving 302 residents from 21 nursing homes, the intervention group reported better overall scores on quality of communication with nursing home staff at 3 months. Family ratings of symptom management and satisfaction with care did not differ between intervention and control groups, and both groups reported high consistency with the resident’s treatment preferences. However, discussions about residents’ preferences guiding treatment were relatively infrequent. In a pretest-posttest pilot study, surrogate decision makers for persons with dementia showed improved quality of communication scores 3 months after viewing the video decision aid. The study also found an increase in the number of palliative care domains addressed in the care plan following the video intervention.

#### Goal of Care Communication Documentation

The most recent study by Volandes et al [35] focused on evaluating GOC documentation in the EHR as the primary outcome [35]. The study used natural language processing–assisted human adjudication to analyze GOC communication documented in the free text of clinical notes. GOC communication encompassed discussions about goals, limitations of life-sustaining treatment, palliative care, hospice, or time-limited trials. The results showed that compared to the usual care arm, the video decision aid arm demonstrated an increased proportion of GOC documentation, along with higher proportions of documented conversations on goals, limitation of life-sustaining treatment, and palliative care. Another study [33] found that the PREPARE group with an advance directive had a higher rate of ACP documentation compared to the control group.

#### Knowledge of Advanced Dementia

Knowledge of advanced dementia was evaluated through various methods. In the study by Volandes et al [37], patient knowledge of advanced dementia was assessed using 5 true or false questions, and participants in the video group showed an increased knowledge score of advanced dementia. Surrogate knowledge was evaluated in the study by Einterz et al [40] using 18 true or false items regarding dementia, GOC, and treatment options. Surrogates demonstrated an increase in the number of correct responses on knowledge-based questions after viewing the decision aid. In addition, in the study by Huang et al [42], persons with dementia and family carers were asked about their knowledge of end-stage dementia treatment and ACP using various questions. The visual aid intervention in this study resulted in significant improvements in knowledge of end-stage dementia treatment and knowledge of ACP among the participants.

#### Decision-Making Performance

Two studies evaluated decision-making performance (decisional skill) and outcome (decisional conflict). Participants’ decisional skills were appraised using a 7-point Likert scale by listening to each sample through headphones by external judges based on the 4 legal standards (Understanding, Expressing a Choice, Reasoning, and Appreciation). With a sample of 20 participants, the pretest-posttest results showed that participants’ overall decision-making performance was significantly different in the 2 experimental conditions, with a large effect size of 4.44 [11]. Personal conflicts about decisions for end-of-life care were measured with a modification of the Decisional Conflict Scale. A 1-group, pretest-posttest, experimental study recruited 40 dyads of persons diagnosed with mild dementia and their family carers. The intervention resulted in significant reductions in decisional conflict [42].

#### Health Care Resource Use

Health care use outcomes included the proportions of hospital transfer, burdensome intervention, hospice care enrollment, late transitions, and do-not-hospitalize (DNH) orders. In the Pragmatic Trial of Video Education in Nursing Homes study, which implemented the video decision aid in nursing homes, there was no significant reduction in hospital transfers, burdensome interventions, or hospice care enrollment between the intervention and control groups [31]. Secondary analyses in subsets of short-stay and long-stay residents with advanced illness also did not show significant differences in hospital transfers or hospice care enrollment [29,30]. However, there was a higher proportion of long-stay residents with new DNH orders in the treatment group compared to the control group, indicating a potential impact on care preferences [30]. In another secondary analysis of 2848 decedents among long-stay residents with advanced illness (923 intervention and 1925 control), there was no statistically significant reduction in the proportion of multiple hospital transfers and late transitions before death [32]. In contrast, there was a statistically significant reduction in the proportion of 90-day hospital transfers in these deceased long-stay residents [32]. Another study recorded health care use outcomes such as do-not-resuscitate orders, DNH orders, completion of a Medical Orders for Scope of Treatment, and the number of hospital or emergency transfers [28]. The intervention group showed higher completion rates of a Medical Orders for Scope of Treatment order set by nursing home physicians or nurse practitioners compared to the control group. In addition, residents in the intervention group were found to be half as likely to experience hospital transfers per 90 person-days compared to the control group during the 9-month follow-up period. These findings suggest that video decision aids may play a role in facilitating new care preferences, such as DNH orders, and in reducing hospital transfers in certain populations of nursing home residents with advanced illness.

### Meta-Analyses

**Figure 2:**
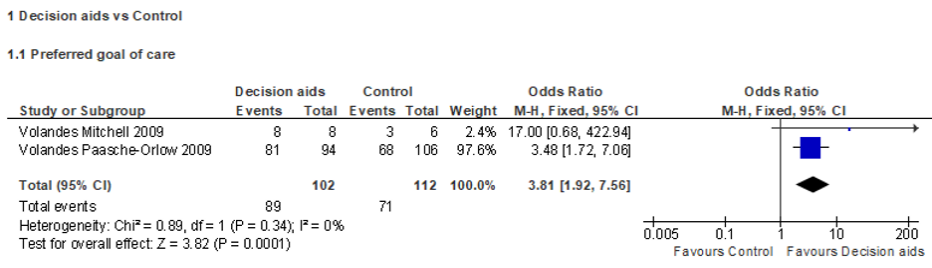
Forest plot for the preferred goal of care (opting for comfort care).

**Figure 3:**
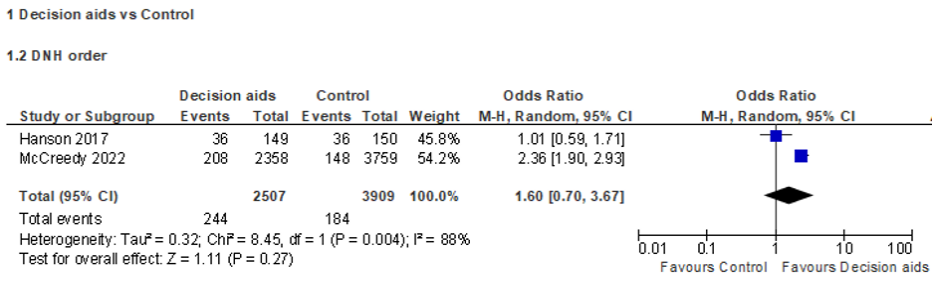
Forest plot for the do-not-hospitalize (DNH) order.

Due to the difference in resident- or family-rated outcome measures and study design, only 4 RCTs were included in the meta-analyses on 2 outcomes (2 RCTs for each outcome). Results on the preferred goal of care (comfort care) from 2 RCTs were extracted and used in the meta-analysis [36,37]. There was a low degree of heterogeneity (*I*^2^=0%; *P*=.34), likely because both studies were conducted by Volandes et al [36,37] in similar settings. In the sample with 102 participants in the intervention and 112 in the control group, the meta-analysis suggested that the video decision aid was effective in increasing the proportion of participants opting for comfort care (OR 3.81, 95% CI 1.92-7.56) compared to the control group (Figure 2). In addition, results on the recorded DNH orders from 2 RCTs were extracted and used in the meta-analysis [28,30]. There was a high degree of heterogeneity (*I*^2^=88%; *P*=.004); therefore, the random effects model was applied. This high heterogeneity might come from the great difference in sample size across these 2 RCTs. In the sample with 2507 participants in the intervention and 3909 participants in the control group, the meta-analysis suggested that the video decision aid was ineffective in the proportion of recorded DNH orders (OR 1.60, 95% CI 0.70-3.67) compared to the control group (Figure 3).

## Discussion

### Principal Findings

The review highlighted the use of technologies to support the development and delivery of decision aids on the internet for palliative and end-of-life dementia care. These digital decision aids mainly focused on the decision about end-of-life GOC, including 3 levels of life-prolonging care, limited care, and comfort care. Narrative synthesis demonstrated that digital decision aids were effective in improving the probability of opting for comfort care [19,36,37]; concordance with the primary GOC between patients, families, and clinicians [28,34]; the quality of end-of-life communication and palliative care [28,40]; knowledge of advanced dementia [37,40]; documentation of GOC communication [35]; and decision-making skills [11], as well as decreasing decisional conflict [42]. Meta-analyses supported that video decision aids were effective in increasing the proportion of opting for comfort care but inconclusive for the proportion of DNH orders. However, these results need to be interpreted with caution as only 2 RCTs were included for meta-analysis of each outcome. Pilot studies examining the feasibility and acceptability of decision aids showed that most participants found these decision aids relevant to their needs, easy to use, and were able to complete the intervention sessions [38-41,43-45]. These findings suggested that decision aids were a feasible and acceptable approach to support decision-making in end-of-life dementia care. However, given the variety of outcome measures used across studies, more consistent outcomes need to be evaluated in large-scale RCTs to provide more robust evidence on the effectiveness of decision aids in people with dementia.

### Research and Clinical Implications

Digital decision aids could be used in routine care to facilitate the shared decision-making process between people with dementia, family carers, and health care providers. The use of technologies in the development and delivery of decision aids offers an opportunity to incorporate flexible applications (eg, texts, animations, images, audios, videos, games, and social networking tools) to provide health information and inform decision-making [47]. Previous systematic evidence suggested that technologies have been inadequately applied in palliative and end-of-life dementia care [48]. Compared to paper-based decision aids, modern and advanced technologies are unique in that they not only deliver the complex information required for decision-making (such as visual aids and videos) but also integrate personalized preferences and clinical conditions into the ultimate decisions (such as web pages and applications). Four formats of technologies, including visual aids, videos, web pages, and telehealth, were developed in the identified decision aids. External visual support (eg, graphs, pictures, and videos) was helpful in visualizing hypothesized situations for the patient and their carer to make the real decision [11,37]. Web pages were designed to involve dementia care networks and enable the patient to give their individual opinions about dementia-related topics and individual circumstances through specific functions and modules [39,43,44]. Furthermore, telehealth can mitigate barriers related to time scheduling and geographic constraints, allowing patients and family carers to engage in health care decision-making conversations from their homes [41]. These technology-based decision aids are supposed to support the development and delivery of decision support on the internet. However, most decision aids are not yet available on the internet, except for the PREPARE website [46]. To benefit the maximum number of people, researchers and developers need to consider publishing their decision support materials (such as videos, modules, and websites) on the web and advocate for the significance of end-of-life conversations. In addition, implementation studies are needed to generate implementation strategies to facilitate the uptake and integration of digital decision aids in clinical practice for quality palliative and end-of-life dementia care.

Decision-making to administer, withhold, or withdraw life-saving treatment at end-of-life needs to be a step-by-step communication process in which patient autonomy must be respected [49]. Decision aids are feasible for patients and their family carers due to the length of time needed to learn the knowledge and the incorporation of personal values and preferences [23]. Many decision aids were designed specifically for patients with advanced dementia and their family carers in long-term care settings (eg, nursing homes and residential care homes). Considering the gradual loss of communicative and cognitive abilities for decision-making, the engagement of family carers is necessary to represent personal and family values. However, earlier identification of dementia as a terminal illness would allow cognitively capable patients to be more actively involved in making decisions about their future end-of-life care. Therefore, earlier implementation of decision aids needs to be developed and delivered to patients with early dementia. In addition, the summary of the decision and related information could be shared with clinicians to implement personal decisions in the clinical treatment based on the patient’s clinical condition. Practically, a follow-up care plan meeting with clinicians after reviewing the decision aid is needed to share the decision. Furthermore, decisional coaching has not been applied in the identified decision aids. It is important to note that sensitive medical decisions around end-of-life care need to be facilitated by professional guidance. A decision coach could facilitate the decision-making process with the decision aid and eventually encourage the decision maker to share their decisions and preferences with significant others and health care providers [50]. Decisional coaching could be applied in the future development of end-of-life decision aids.

### Strengths and Limitations

This systematic review was conducted with methodological rigor. We conducted a thorough search and independent full-text screening and quality appraisal. We also included both RCTs and pilot studies to comprehensively explore digital decision aids. Therefore, the narrative synthesis allowed for the comprehensive inclusion of various technologies and various outcome measures. However, due to the differences in outcome measures and study design, only 4 RCTs testing video decision aids were used in the meta-analyses. Therefore, there was a lack of power to support a conclusive finding regarding the effect of video decision aids. Large-scale RCTs are needed to evaluate the effect of these decision aids on GOC concordance, quality of palliative care, decision-making performance, and health care use outcomes. In addition, due to the lack of comprehensive translation capabilities for non-English publications, the restriction to English-language studies is acknowledged as a limitation. Furthermore, while the included studies evaluated the effectiveness of digital decision aids in dementia, the challenges and related strategies to implement these tools in real-world settings have not been explored and synthesized. Future studies need to address contextual factors such as clinician workflows, health literacy, and cost-effectiveness to better introduce and implement these tools to the public.

### Conclusions

Digital decision aids offer a feasible and acceptable approach to support the shared decision-making between patients, families, and clinicians. The included studies reported various outcome measures in preferred GOC, quality of palliative care, decision-making performance, and health care use. Although current evidence supported the benefits of digital decision aids in the proportion of opting for comfort care, more large-scale RCTs are needed to include consistent outcome measures to evaluate the effect of digital decision aids for palliative and end-of-life dementia care. Future development and implementation of digital decision aids needs to engage patients with early dementia and family carers in the community rather than only focusing on patients with advanced dementia residing in long-term care settings. Finally, a summary of the end-of-life decision can be shared with clinicians who provide direct caring services to patients with dementia or family carers to facilitate the shared decision-making process for palliative dementia care.

## Data Availability

All data generated or analyzed during this study are included in this published article.

## Appendix

Multimedia Appendix 1 PRISMA 2020 checklist.

Multimedia Appendix 2 Search strategy.

Multimedia Appendix 3 Results of the quality appraisal.

## Abbreviations

ACP: advance care planning
DNH: do-not-hospitalize
EHR: electronic health record
GOC: goals of care
OR: odds ratio
PRISMA: Preferred Reporting Items for Systematic Reviews and Meta-Analyses
RCT: randomized controlled trial
