# Supplementary material for: Digital Decision Aids to Support Decision-Making in Palliative and End-of-Life Dementia Care: Systematic Review and Meta-Analysis
Source: J Med Internet Res. 2025 Jun 23;27:e71479. doi: 10.2196/71479 (PMC12235206; doi:10.2196/71479)
Supplement: Multimedia Appendix 2 [file jmir_v27i1e71479_app2.docx]

**Appendix 1**

**Search Strategy**

Database: PubMed, EMBASE, CINAHL, Web of Science

| **1. PubMed** | |
| --- | --- |
| #1 | internet OR digital OR technology OR technologies OR visual OR web OR video OR audio OR media OR computer OR tablet OR electronic OR telecommunication OR telemedicine OR telephone OR television OR text messaging OR videoconferencing OR mobile phone |
| #2 | informed choice OR decision-support* OR decision aids OR decision tree OR decision-mak* OR informed decision |
| #3 | ((((end of life[Title/Abstract] OR eol[Title/Abstract] OR terminal care[Title/Abstract] OR palliative care[Title/Abstract] OR advance care planning[Title/Abstract] OR hospice care[Title/Abstract]) OR (palliative care[MeSH Terms])) OR (hospice care[MeSH Terms])) OR (advance care planning[MeSH Terms])) OR (end of life care[MeSH Terms]) |
| #4 | (((memory disorder*[Title/Abstract] OR cognition[Title/Abstract] OR dementia[Title/Abstract] OR Alzheimer[Title/Abstract] OR dement*[Title/Abstract] OR cogni*[Title/Abstract]) OR (memory disorder[MeSH Terms])) OR (cognition disorders[MeSH Terms])) OR (dementia[MeSH Terms]) |
| #5 | #1 AND #2 AND #3 AND #4 |
|  | Items found: 369 |

| **2. EMBASE** | |
| --- | --- |
| #1 | (internet or digital or technology or technologies or visual or web or video or audio or media or computer or tablet or electronic or telecommunication or telemedicine or telephone or television or text messaging or videoconferencing or mobile phone).mp. |
| #2 | (informed choice or decision-support* or decision aids or decision tree or decision-mak* or informed decision).mp. |
| #3 | (end of life or eol or terminal care or palliative care or advance care planning or hospice care).mp. |
| #4 | (memory disorder* or cognition or dementia or Alzheimer or dement* or cogni*).mp. |
| #5 | 1 and 2 and 3 |
|  | Items found: 403 |

| **3. CINAHL Plus** | |
| --- | --- |
| #1 | TX (internet OR digital OR technology OR technologies OR visual OR web OR video OR audio OR media OR computer OR tablet OR electronic OR telecommunication OR telemedicine OR telephone OR television OR text messaging OR videoconferencing OR mobile phone) |
| #2 | TX (informed choice OR decision-support* OR decision aids OR decision tree OR decision-mak* OR informed decision) |
| #3 | AB (end of life OR eol OR terminal care OR palliative care OR advance care planning OR hospice care) |
| #4 | AB (memory disorder* OR cognition OR dementia OR Alzheimer OR dement* OR cogni*) |
| #5 | #1 AND #2 AND #3 AND #4 |
|  | Items found: 25 |

| **4. Web of Science** | |
| --- | --- |
| #1 | internet OR digital OR technology OR technologies OR visual OR web OR video OR audio OR media OR computer OR tablet OR electronic OR telecommunication OR telemedicine OR telephone OR television OR text messaging OR videoconferencing OR mobile phone (All Fields) |
| #2 | informed choice OR decision-support* OR decision aids OR decision tree OR decision-mak* OR informed decision (All Fields) |
| #3 | end of life OR eol OR terminal care OR palliative care OR advance care planning OR hospice care (Topic) |
| #4 | memory disorder* OR cognition OR dementia OR Alzheimer OR dement* OR cogni* (Topic) |
| #5 | #1 AND #2 AND #3 |
|  | Items found: 477 |
