# Supplementary material for: Digital Decision Aids to Support Decision-Making in Palliative and End-of-Life Dementia Care: Systematic Review and Meta-Analysis
Source: J Med Internet Res. 2025 Jun 23;27:e71479. doi: 10.2196/71479 (PMC12235206; doi:10.2196/71479)
Supplement: Multimedia Appendix 3 [file jmir_v27i1e71479_app3.docx]

**Cochrane Risk of Bias (RoB V2)**

|  | Random sequence generation (selection bias) | Allocation concealment (selection bias) | Blinding of participants and personnel (performance bias) | Blinding of outcome assessment (detection bias) | Incomplete outcome data (attrition bias) | Selective reporting (reporting bias) | Other bias |
| --- | --- | --- | --- | --- | --- | --- | --- |
| Volandes (2023) | Low | Low | Low | Low | Low | Low | Low |
| Towsley (2022) | Low | Unclear | High | Low | Low | Low | Low |
| Moyo (2022) | Low | Low | Low | Low | Low | Low | Low |
| McCreedy (2022) | Low | Low | Low | Low | Low | Low | Low |
| Loomer (2021) | Low | Low | Low | Low | Low | Low | Low |
| Mitchell (2020) | Low | Low | Low | Low | Low | Low | Low |
| Sudore (2018) | Low | Low | Low | Low | Low | Low | Low |
| Hanson (2017) | Low | Low | Low | Low | Low | Low | Low |
| Volandes (2009a) | Low | Low | High | High | Low | Low | Low |
| Volandes (2009b) | Low | Low | High | High | Low | Low | Low |

Low risk of bias, Unclear risk of bias, High risk of bias

**Risk of Bias in Non-Randomized Studies of Interventions, Version 2 (ROBINS-I V2)**

|  | Confounding | Selection of participants into the study | Classification of interventions | Deviations from intended interventions | Missing data | Measurement of the outcome | Selection of the reported result |
| --- | --- | --- | --- | --- | --- | --- | --- |
| Gabbard (2024) | Low | Low | Low | Low | Low | Low | Low |
| Cardona (2022) | Low | Moderate | Low | Unclear | Low | Low | Low |
| Behrens (2022) | Low | Moderate | Low | Unclear | Low | Low | Low |
| Kotwal (2021) | Low | Low | Low | Low | Low | Low | Low |
| Towsley (2020) | Low | Low | Low | Low | Low | Low | Low |
| Huang (2020) | Low | Low | Low | Low | Low | Low | Low |
| Chang (2020) | Low | Low | Low | Low | Low | Low | Low |
| Span (2015) | Low | Moderate | Low | Unclear | Low | Low | Low |
| Einterz (2014) | Low | Low | Low | Low | Low | Low | Low |
| Volandes (2007) | Low | Low | Low | Low | Low | Low | Low |

Low risk of bias, Moderate risk of bias, Serious risk of bias, and Critical risk of bias
